# Pregnancy outcomes related to the treatment of sarcomas with anthracyclines and/or ifosfamide during pregnancy

**DOI:** 10.1002/cam4.4707

**Published:** 2022-03-28

**Authors:** Devon Miller, John A. Livingston, Yeonhee Park, Kristi Posey, Sonia Godbole, Keith Skubitz, Steven I. Robinson, Mark Agulnik, Lara E. Davis, Brian A. Van Tine, Angela C. Hirbe, Amanda Parkes

**Affiliations:** ^1^ University of Wisconsin‐Madison School of Medicine and Public Health Madison Wisconsin USA; ^2^ MD Anderson Cancer Center Houston Texas USA; ^3^ Department of Biostatistics and Medical Informatics, University of Wisconsin‐Madison Madison Wisconsin USA; ^4^ Washington University in St. Louis School of Medicine St. Louis Missouri USA; ^5^ University of Minnesota Minneapolis Minnesota USA; ^6^ Mayo Clinic Rochester Minnesota USA; ^7^ City of Hope Duarte California USA; ^8^ Oregon Health & Science University Portland Oregon USA

## Abstract

**Background:**

Sarcomas are rare diagnoses but are seen with relative frequency in adolescents and young adults and thus can present in pregnancy. We sought to study the administration of anthracyclines and/or ifosfamide in pregnancy‐associated sarcomas.

**Patients and Methods:**

We conducted a multi‐institutional retrospective study, identifying sarcoma patients who received anthracyclines and/or ifosfamide during pregnancy. Chart review identified variables related to demographics, cancer diagnosis, therapies, and outcome of the patient and fetus. Wilcoxon rank‐sum test compared two independent samples.

**Results:**

We identified 13 patients at seven institutions with sarcoma who received anthracyclines and/or ifosfamide during pregnancy, including four bone sarcomas and nine soft tissue sarcomas diagnosed at a mean gestational age of 16.7 ± 5.9 weeks. Only nine patients had live births (9/13, 69.2%), with mean gestational age of 30.8 ± 3.8 weeks at delivery. The four patients with pregnancy loss all received both doxorubicin and ifosfamide, with chemotherapy initiated at 15.5 weeks as compared with 21.3 weeks for those patients with live births (*p* = 0.016).

**Conclusion:**

In this multi‐institutional study of sarcoma chemotherapy regimens administered during pregnancy, we found a high rate of fetal demise that was seen only in patients receiving both doxorubicin and ifosfamide and statistically more likely with chemotherapy initiation earlier in the second trimester. While limited by a small sample size, our study represents the largest study of sarcoma patients that received anthracyclines and/or ifosfamide in pregnancy thus far reported and supports development of an international registry to study concerns raised by our study.

## Graphical abstract

In this multi‐institutional study of anthracyclines and/or ifosfamide in pregnancy‐associated sarcomas, we characterized the largest group of sarcoma patients receiving such treatment in pregnancy thus far reported and identified a high rate of pregnancy complications and fetal demise. Fetal deaths were only seen in patients who received both doxorubicin and ifosfamide and were statistically more likely with chemotherapy initiation earlier in the second trimester. Maternal cancer‐related outcomes showed chemotherapy response or stable disease in the majority of patients with a median disease‐free survival of 62 months.

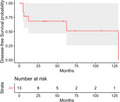

## 1 INTRODUCTION

While rare diagnoses, sarcomas are seen with relative frequency in adolescents and young adults and thus can present in pregnancy.^1^, ^2^ Approximately one in 1000–2000 pregnancies are complicated by cancer, with the most common pregnancy‐associated cancers represented by those malignancies that occur during a female's peak reproductive years (e.g., breast cancer, cervical cancer, hematological malignancies, and melanoma).^3^, ^4^ As such, there is limited data on pregnancy‐associated sarcomas. However, data are available to show that almost all types of sarcomas have been seen in pregnancy, most commonly soft tissue sarcomas such as leiomyosarcoma and liposarcoma as well as bone sarcomas such as osteosarcoma and Ewing's sarcoma.^5^

While a sarcoma diagnosis during pregnancy represents a vulnerable time for mother and fetus, there is limited sarcoma‐specific data to support safety of commonly used sarcoma chemotherapy regimens in pregnancy. The majority of standard sarcoma treatment algorithms involve use of anthracyclines, thus allowing for the extrapolation of safety data of anthracyclines in pregnancy‐associated breast cancers and hematologic malignancies.^6^, ^7^ This data supports the safety of anthracyclines except idarubicin when given during the second and third trimesters and allows pregnant patients to still receive guideline concordant therapy.^8^, ^9^ Despite overlapping use of anthracyclines, however, sarcoma treatment algorithms often involve addition of other agents less well studied in pregnancy and involve relatively high doses of systemic therapy agents, including higher doses of anthracyclines. While certain agents used in the treatment of sarcomas are known to be contraindicated, such as the absolute contraindication in pregnancy for methotrexate, safety of other commonly used agents such as ifosfamide in pregnancy is less well established and limited to case reports and single‐center series.^10^, ^11^, ^12^, ^13^, ^14^, ^15^, ^16^, ^17^, ^18^ Additionally, there is limited data to support maternal outcomes of pregnancy‐associated sarcomas, which is critical in considering the delicate balance of maternal and fetal well‐being in the treatment of pregnancy‐associated malignancies.

Given the need for multicenter data to study maternal and fetal outcomes related to the treatment of pregnancy‐associated sarcomas, using standard treatment regimens at traditional doses used in the treatment of sarcomas, we undertook a multi‐institutional retrospective study to study the administration of anthracyclines and/or ifosfamide in pregnancy‐associated sarcomas, characterizing the largest group of sarcoma patients receiving such treatment in pregnancy thus far reported.

## 2 MATERIALS AND METHODS

### 2.1 Medical record review

We conducted a multi‐institutional retrospective study to identify patients with bone or soft tissue sarcoma who received anthracyclines and/or ifosfamide during pregnancy. Patients were identified by providers at their own institutions after being contacted by the principal investigator. Sarcoma‐specific providers at academic institutions were approached via email and were given up to 8 weeks to respond, with one email reminder sent. Ultimately, patients were identified by providers at seven institutions (University of Wisconsin, MD Anderson Cancer Center, Washington University in St. Louis, City of Hope, University of Minnesota, Mayo Clinic, and Oregon Health & Science University). Retrospective chart review identified variables from the patient's chart related to demographics, cancer diagnosis, and therapies including those received during pregnancy, and outcome of the patient and fetus. Pregnancy complications and fetal outcomes were identified through review of the maternal medical record only. The study was conducted in accordance with all relevant guidelines and procedures and was approved by the University of Wisconsin Institutional Review Board.

### 2.2 Statistical analysis

Data were summarized numerically using descriptive statistics such as proportion for categorical variables and mean (standard deviation) or median (range with minimum and maximum) for continuous variables. We performed Wilcoxon rank‐sum test to compare two independent samples (i.e., patients with pregnancy loss and patients with live birth). Survival times were displayed graphically using Kaplan–Meier curves with median survivals.

## 3 RESULTS

We identified 13 patients from seven institutions with sarcoma who received anthracyclines and/or ifosfamide during pregnancy, including four pregnancy‐associated bone sarcomas (4/13, 30.8%) and nine pregnancy‐associated soft tissue sarcomas (9/13, 69.2%) diagnosed at a mean gestational age of 16.7 ± 5.9 weeks. The clinical features of the patients with pregnancy‐associated sarcomas are presented in Table 1. Three patients (3/13, 23.1%) were G1P0, while the remaining 10 patients had prior pregnancies. Eleven pregnancies (11/13, 84.6%) were conceived naturally and the remaining two pregnancies were from intrauterine insemination (IUI, *n* = 1) and in vitro fertilization (IVF, *n* = 1). Mean age at conception for all patients was 28.5 ± 4.4 years and only one patient was considered advanced maternal age with age at time of conception of 35 years.

**TABLE 1:** Baseline characteristics of patients with pregnancy‐associated sarcomas (*n* = 13)

Variable	
Patient age at sarcoma diagnosis (years)	Mean (SD) 28.54 (4.41)
Race	*n* (%)
American Indian or Alaska Native	0 (0)
Asian	0 (0)
Black or African American	1 (7.7)
White or Caucasian	9 (69.2)
Other	1 (7.7)
Unknown/Refuse to Answer	2 (15.4)
Ethnicity	*n* (%)
Hispanic	2 (15.4)
Non‐Hispanic	11 (84.6)
Time to diagnosis from onset of symptoms (months)	Mean (SD) 4.58 (5.75)
Stage at diagnosis	*n* (%)
Localized	10 (76.9)
Metastatic	3 (23.1)
Location of primary tumor	*n* (%)
Extremity	5 (38.5)
Lung	2 (15.4)
Chest wall	2 (15.4)
Pelvis	2 (15.4)
Abdominal wall	1 (7.7)
Mandible	1 (7.7)
Size of primary tumor at diagnosis (cm)	Mean (SD) 11.4 (7.31)
Diagnosis grouping	*n* (%)
Bone sarcoma	4 (30.8)
Soft tissue sarcoma	9 (69.2)
Histology	*n* (%)
*Soft tissue sarcomas*	
Malignant peripheral nerve sheath tumor	1 (7.7)
Synovial sarcoma	1 (7.7)
Myxofibrosarcoma	1 (7.7)
High‐grade spindle cell sarcoma	1 (7.7)
Desmoid tumor	1 (7.7)
Unclassified small cell malignancy	1 (7.7)
High‐grade spindle cell sarcoma	2 (15.4)
Embryonal rhabdomyosarcoma	1 (7.7)
*Bone sarcomas*	
Osteosarcoma	3 (23.1)
Ewing's sarcoma	1 (7.7)
Type of pregnancy	*n* (%)
Natural	11 (84.6)
In vitro fertilization	1 (7.7)
Intrauterine insemination	1 (7.7)
Age of mother at conception (years)	Mean (SD) 28.5 (4.4)
Gestational age at diagnosis (weeks)	Mean (SD) 16.7 (5.9)

As seen in Table 2, all patients received anthracycline chemotherapy during pregnancy (13/13, 100%). Anthracyclines were most commonly given in combination with ifosfamide, with nine patients (9/13, 69.2%) receiving a regimen with both agents during pregnancy. Twelve patients received doxorubicin (12/13, 92.3%) and one patient received epirubicin 60 mg/m^2^. Doxorubicin was administered at doses of 60 mg/m^2^ to 75 mg/m^2^, with nine patients (9/12, 75.0%) receiving the 75 mg/m^2^ doxorubicin dosing per cycle. Ifosfamide was administered at doses of 7.5–10 g/m^2^ per cycle, with only two patients (2/9, 22.2%) receiving the 7.5 g/m^2^ ifosfamide dosing per cycle. The majority of chemotherapy regimens followed standard regimens for the histologic subtypes,^6^, ^7^ however methotrexate was not given for osteosarcomas given this is a known abortifacient and teratogen.^19^, ^20^ One patient with pathology reported as unclassified small cell malignancy received two different chemotherapy regimens during pregnancy, initially receiving doxorubicin/cyclophosphamide that was switched at a gestational age of 29 weeks to doxorubicin/ifosfamide due to progression. Chemotherapy was initiated at a mean gestational age of 19.5 ± 4.0 weeks (range 13–27 weeks) with only one patient starting chemotherapy during the third trimester and the remainder starting chemotherapy during the second trimester. Patients received an average of three cycles of chemotherapy during pregnancy with a total of 43 cycles of chemotherapy regimens administered during pregnancy for the 13 patients in the study. Notably, of the 10 patients with localized sarcomas who were treated with curative intent, eight received neoadjuvant chemotherapy (8/10, 80.0%), while the remaining two patients received adjuvant chemotherapy (2/10, 20.0%) during pregnancy. In addition to their receipt of systemic therapy during pregnancy, two patients also underwent surgery during pregnancy, both with R0 resection of localized extremity tumors occurring at 19‐ and 36‐weeks gestational age.

**TABLE 2:** Characteristics and pregnancy outcomes of patients with pregnancy‐associated sarcomas (*n* = 13)

Patient No.	Histology	Type of pregnancy	Systemic therapy during pregnancy	Gestational week systemic therapy started (weeks)	Pregnancy complications	Live fetal birth?	Gestational age at birth or fetal demise (weeks)
1	Malignant peripheral nerve sheath tumor	Natural	Epirubicin/Ifosfamide	24	Fetal growth restriction	Yes	26
2	Osteosarcoma	Natural	Doxorubicin/Ifosfamide	22	Fetal growth restriction, anhydramnios, maternal hemoperitoneum	Yes	27
3	Synovial sarcoma	In vitro fertilization	Doxorubicin/Ifosfamide	16	Fetal growth restriction, anhydramnios, intrauterine fetal demise	No	20
4	Osteosarcoma	Natural	Doxorubicin/Cisplatin	21	None	Yes	36
5	Ewing sarcoma	Natural	Vincristine, Doxorubicin, Cyclophosphamide alternating with Ifosfamide and Etoposide	16	Fetal growth restriction, fetal demise found at time of induction for abnormal umbilical artery Doppler study	No	24
6	Myxofibrosarcoma	Natural	Doxorubicin/Ifosfamide	22	Reduced fetal movements, abnormal intrauterine fetal heart rates	Yes	26
7	High‐grade spindle cell sarcoma	Natural	Doxorubicin/Ifosfamide	13	Intrauterine stillbirth	No	16
8	Osteosarcoma	Natural	Doxorubicin/Cisplatin	23	None	Yes	34
9	Desmoid	Natural	Doxorubicin	16	Fetal growth restriction	Yes	33
10	Unclassified small cell malignancy	Natural	Doxorubicin/Cyclophosphamide ‐>Doxorubicin/Ifosfamide	18	Fetal growth restriction, asymmetric growth restriction	Yes	32
11	High‐grade spindle cell sarcoma	Natural	Doxorubicin/Ifosfamide	27	Oligohydramnios	Yes	30
12	High grade spindle cell sarcoma	Intrauterine insemination	Doxorubicin/Ifosfamide	17	Fetal demise	No	23
13	Embryonal rhabdomyosarcoma	Natural	Vincristine/Doxorubicin/Cyclophosphamide	19	None	Yes	34

As seen in Table 2, the majority of patients had pregnancy complications (10/13, 76.9%), most commonly fetal growth restriction, which was seen in six patients (6/13, 46.2%). Of the 13 patients in our study, nine had live births (9/13, 69.2%). Prematurity was observed in all cases, with mean gestational age of 30.8 ± 3.8 weeks at time of delivery (range 26–36 weeks) and 66.7% (6/9) of infants required a stay in the neonatal intensive care unit (NICU). Median birth weight was 1119.1 grams (range: 660.5–2020 g), with no infants with available birth weight above the 50th percentile for birth weight for gestational age. Only one patient delivered vaginally, with all other patients undergoing cesarean delivery. Of the four patients with fetal demise, the average gestational age at time of fetal demise was 20.8 ± 3.1 weeks (range 16–24 weeks). The four patients with pregnancy loss all received both doxorubicin and ifosfamide, with ifosfamide dosing of 9 to 10 g/m^2^ per cycle. Neither of the two patients who received ifosfamide 7.5 g/m^2^ per cycle dosing experienced fetal loss. Notably, patients with pregnancy loss had chemotherapy initiated at 15.5 weeks of gestation as compared with chemotherapy initiation at 21.3 weeks of gestation for those patients with live birth, which was a statistically significant finding based on Wilcoxon rank‐sum test (*p* = 0.016).

**FIGURE 1:**
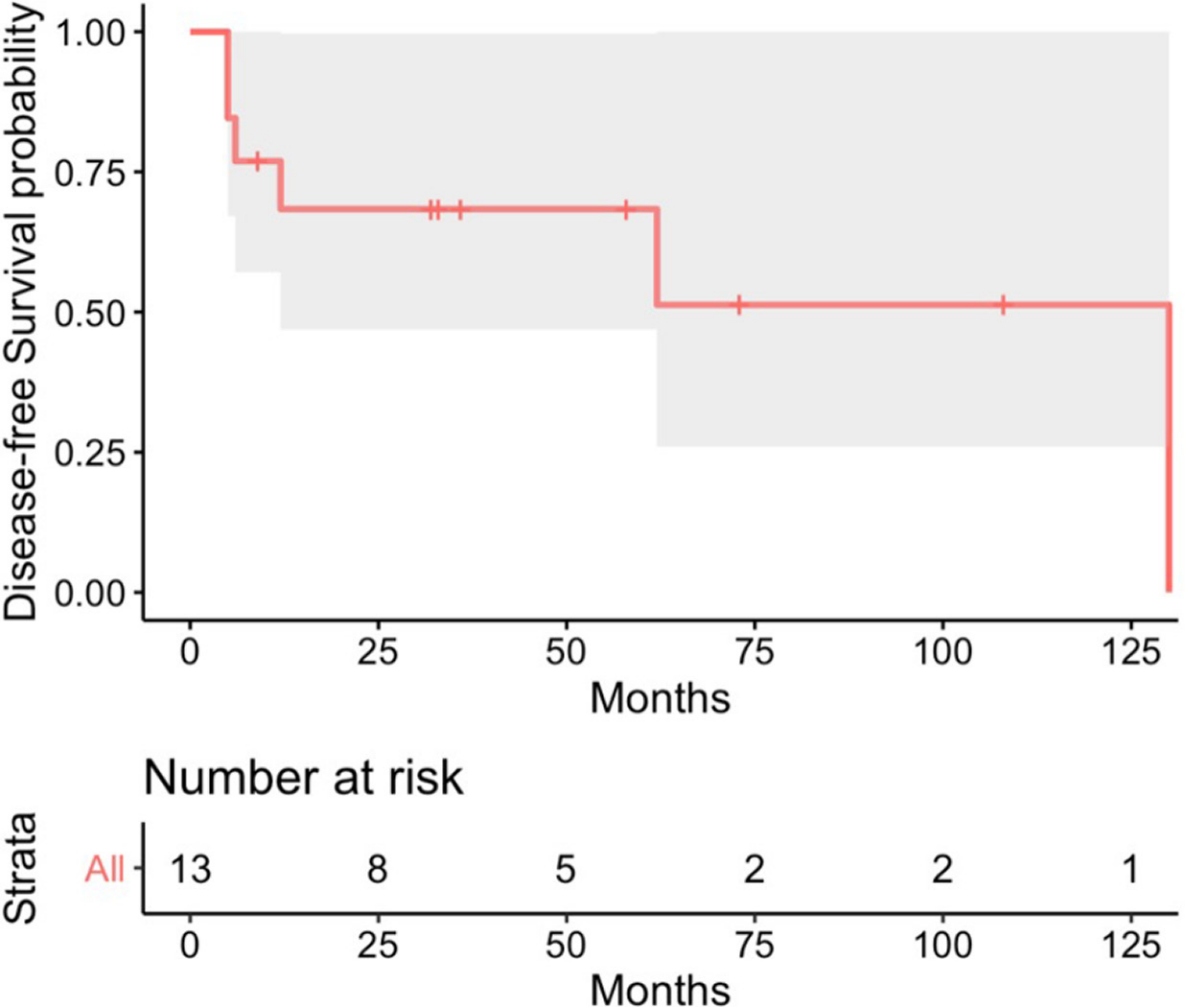
Kaplan–Meier estimates of disease‐free survival time curve (*n* = 13) with 95% confidence interval represented by shaded area

Maternal cancer‐related outcomes showed only two cases of progressive disease in response to chemotherapy given during pregnancy (2/13, 15.4%). Of the remaining 11 patients, one had a complete response (1/13, 8.3%), five had partial response (5/13, 41.7%), and five had stable disease (5/13, 41.7%) in response to chemotherapy given during pregnancy. The Kaplan–Meier estimate of disease‐free survival (DFS) is seen in Figure 1, with median DFS of 62 months. Three patients in our study died (3/13, 23.1%), all due to progressive disease, with median time from pregnancy to death of 9 months (range: 5–17 months) and median time from diagnosis to death of 4 months (range: 4–12 months). No cases of cardiomyopathy were identified in our study.

Considering the three maternal deaths in further detail, all three patients had soft tissue sarcomas (3/3, 100%) diagnosed at a mean patient age of 31.7 ± 2.9 years and a mean gestational age of 18.0 ± 4.4 weeks. All three patients (3/3, 100%) received palliative intent chemotherapy during pregnancy with doxorubicin 75 mg/m^2^ plus ifosfamide 10 g/m^2^. Chemotherapy was initiated at a mean gestational age of 20.7 ± 5.5 weeks. Two of the patients (2/3, 66.7%) had progressive disease as their best response to chemotherapy given during pregnancy, while one patient had partial response as their best response to chemotherapy given during pregnancy.

## 4 DISCUSSION

Pregnancy‐associated malignancies are associated with risk to both the mother and fetus, thus necessitating availability of data to support the safety of cancer‐directed therapies in pregnancy. In this multi‐institutional study, we report the largest group of sarcoma patients receiving anthracyclines and/or ifosfamide during pregnancy, thus filling a substantial gap in our understanding of how to treat these vulnerable patients with pregnancy‐associated sarcomas. Notably, we found a higher rate of pregnancy complications and fetal demise than reported for better studied pregnancy‐associated cancers such as breast cancer and Hodgkin lymphoma,^6^, ^12^ with fetal deaths in our study all seen in patients receiving a combination of doxorubicin and ifosfamide during pregnancy. Strikingly, of the nine patients who received doxorubicin/ifosfamide during pregnancy, only five (5/9, 55.6%) had live births. Comparatively, all patients treated with anthracycline‐based regimens without ifosfamide had live births (4/4, 100%). Interestingly, the outcomes we found in this multi‐institutional study were also poorer than that previously reported in a single‐institution series of patients with pregnancy‐associated sarcoma treated with neoadjuvant doxorubicin/ifosfamide.^18^ Comparing the two studies, it is important to note that the Mir et al. study analyzed patients treated with lower doses of doxorubicin and ifosfamide and that these agents were administered at a later gestational age.

Considering timing of chemotherapy initiation, we found that gestational age at time of chemotherapy initiation was statistically significant between patients with live birth versus pregnancy loss. Specifically, the four patients with pregnancy loss initiated a chemotherapy regimen including both doxorubicin and ifosfamide at a mean of 15.5 weeks of gestation, as compared with chemotherapy initiation at a mean of 21.6 weeks of gestation for those patients with live births (*p* = 0.016). This data suggest that combination therapies with doxorubicin and ifosfamide may have higher risks of fetal harm when given early in the second trimester as compared with later in pregnancy, which would be a unique finding given other data to support the safety of chemotherapy regimens to treat other cancer types in the second trimester or later.^21^, ^22^, ^23^ With regards to the implications of the dosing differences seen between our study and the Mir et al. study of neoadjuvant doxorubicin/ifosfamide given to patients with high‐grade sarcoma in pregnancy, we found that the four patients with pregnancy loss also received a higher average total dose of ifosfamide during pregnancy at 28.3 g/m^2^ (range: 20–45 g/m^2^) compared to 17.1 g/m^2^ (range: 15–20 g/m^2^) for those without pregnancy loss. Notably, patients also received a higher dose of doxorubicin during pregnancy in our study, however, there is data available to support the safety of anthracyclines in the second trimester of pregnancy or later.^24^, ^25^, ^26^, ^27^

Potential confounding factors for the relatively high rate of observed fetal demise seen in our study include traditional pregnancy loss factors such as advanced maternal age and assisted reproductive technology (ART). While reported pregnancy loss rates via natural conception have reported to range from 10% to 16%, pregnancy loss rate with ART have been shown to be higher with reported rates ranging from 20% to 52.6% depending on maternal age.^28^, ^29^, ^30^, ^31^ Notably, both of the patients that conceived via ART (one patient with IVF and one patient with IUI) experienced pregnancy loss. While this is certainly an important consideration, even if these two patients with pregnancy via ART were removed, the remaining 11 patients with natural pregnancies would still have a high rate of pregnancy complications (8/11, 72.7%) and fetal demise (2/11, 18.2%). While the effect of advanced maternal age on pregnancy loss is well known and previously reported, our single patient considered advanced maternal age with age at time of conception of 35 years had a live birth at 30 weeks.^28^, ^31^ In addition to the potential contribution of traditional pregnancy loss factors, consideration must also be made for disease status affecting the pregnancy outcomes seen in our study. Specifically, patients who received ifosfamide in our study were more likely to have a more advanced stage at diagnosis, with median stage III disease seen in patients with anthracycline plus ifosfamide administration as compared with median stage II disease seen in patients who did not receive ifosfamide.

Maternal deaths seen in our study were all due to progressive disease in patients who were receiving palliative intent chemotherapy during pregnancy. There was no significant difference in mean gestational age at time of chemotherapy initiation for patients who died (20.7 ± 5.5 weeks) versus those who were alive at time of last follow‐up (19.2 ± 4.0 weeks), suggesting that maternal deaths were unlikely to be related to delayed treatment initiation. Maternal deaths were also unlikely to be related to undertreatment as all three patients who died received combination therapy with doxorubicin 75 mg/m^2^ and ifosfamide 10 g/m^2^. Patients who died did have a very short mean duration of response to chemotherapy administered during pregnancy (1.7 ± 0.6 months) suggesting aggressive biology, however it is impossible to delineate if pregnancy affected this biology given all patients in our study were diagnosed with sarcoma during pregnancy.

Limitations of our study include the small sample size, although given the rarity of a sarcoma diagnosis during pregnancy and the multi‐institutional nature of our study, this is felt to be a representative population. Most importantly, the small sample size limited our ability to conduct multivariate analysis. In addition to the retrospective nature of our study, our review was limited to the maternal electronic record. These limitations restrict the conclusions that can be made on the role of ifosfamide in fetal outcomes and complications when used during pregnancy.

To our knowledge, this is the largest study of the administration of doxorubicin and/or ifosfamide in pregnancy‐associated sarcomas. Despite its limitations, our study supports development of an international registry to study the concern for increased risk of combination doxorubicin and ifosfamide administration in pregnancy, particularly when given at an earlier gestational age.

## CONFLICT OF INTEREST

The authors declare no conflict of interest.

## AUTHOR CONTRIBUTIONS

DM, JAL, KS, SIR, MA, BAVT, ACH, and AP contributed to study design. DM, KP, SG, KS, SIR, MA, LED, ACH, and AP contributed to data collection. YP conducted statistical analysis. All coauthors participated in the data interpretation and approved the version to be published and agreed to be accountable for all aspects of the work and ensure that questions related to the accuracy or integrity of any part of the work are appropriately investigated and resolved.

## ETHICS STATEMENT

The study was conducted in accordance with all relevant guidelines and procedures and was approved by the University of Wisconsin Institutional Review Board.

## Data Availability

The data that support the findings of this study are available from the corresponding author upon reasonable request.
